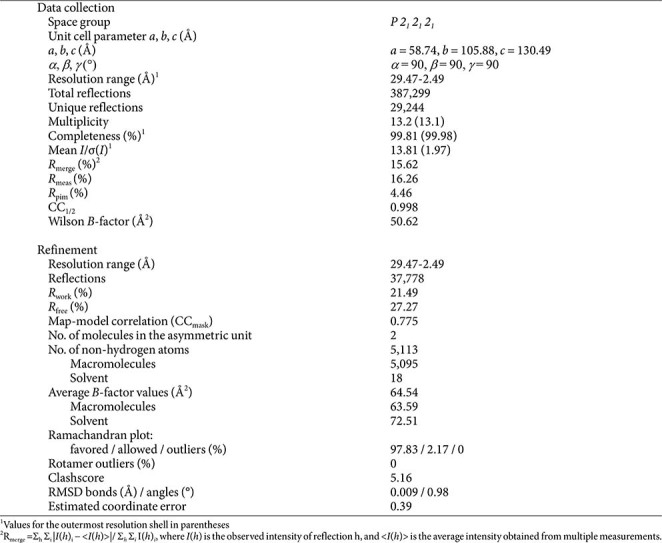# Structural Insights into MltC from *Acinetobacter baumannii*: Conservation of the Catalytic Residue and Flexibility in Substrate Recognition

**DOI:** 10.4014/jmb.2511.11019

**Published:** 2026-01-22

**Authors:** Hyunseok Jang, Chang Min Kim, Hyun Ho Park

**Affiliations:** 1 College of Pharmacy, Chung-Ang University, Seoul 06974, Republic of Korea; 2 Department of Molecular Genetics, University of Texas Southwestern Medical Center, Dallas, TX 75390, USA

**Keywords:** *Acinetobacter baumannii*, Crystal structure, Lytic transglycosylase, MltC

## Abstract

Lytic transglycosylases (LTs) are key enzymes involved in bacterial peptidoglycan remodeling. Here, we present the crystal structure of MltC from *Acinetobacter baumannii* (AbMltC), representing the second reported MltC structure after that of *Escherichia coli* (EcMltC). The AbMltC structure reveals a conserved catalytic residue, E224, equivalent to E217 of EcMltC, which directly participates in glycosidic bond cleavage. Notably, the substrate-binding residue R234, corresponding to R227 of EcMltC, is conserved in sequence but exhibits multiple conformations in AbMltC. This conformational heterogeneity suggests structural flexibility in substrate recognition and provides the structural insights consistent with prior hypothesis that R234 (R227 in EcMltC) functions as a molecular ratchet, facilitating processive cleavage.

## Introduction

The bacterial cell wall, primarily composed of peptidoglycan, provides essential structural support and protection from environmental stress [1, 2]. Remodeling of this macromolecular network is indispensable for bacterial growth, division, and adaptation [3, 4]. The cell wall expansion and remodeling is mediatd by a diverse set of enzymes, including penicillin binding proteins (PBPs), various hydrolases such as amidases and endopeptidases, and a diverse set of lytic transglycosylases (LTs) [5]. LTs catalyze the non-hydrolytic cleavage of glycan strands, producing 1,6-anhydromuramyl termini as signature products of the reaction [6, 7]. Through their activities, LTs not only contribute to cell wall turnover but also participate in processes such as peptidoglycan recycling, cell division, and the assembly of macromolecular complexes including flagella and secretion systems [7, 8]. LTs are classified into several families based on their structural characteristics. Among them, the MltC subfamily is particularly distinguished by specific domain organization. Consequently, *Escherichia coli* MltC (EcMltC), the most extensively characterized member of this group, has served as the structural prototype for understanding this enzyme family [7, 9].

*A. baumannii* is an opportunistic Gram-negative pathogen that has emerged as a leading cause of hospital-acquired infections, particularly ventilator-associated pneumonia, bloodstream infections, and wound infections [10]. Its remarkable ability to acquire resistance determinants has led to widespread multidrug resistance, making *A. baumannii* a critical-priority pathogen according to the World Health Organization [11]. The bacterial cell wall is a key determinant of both survival and antibiotic resistance, as it provides a protective barrier against host immune defenses and antibacterial agents. Enzymes involved in peptidoglycan metabolism, including LTs, are therefore not only fundamental to bacterial physiology but also represent potential targets for therapeutic intervention.

In this study, we determined the crystal structure of MltC from *A. baumannii* (AbMltC), representing the second reported MltC structure after MltC from *Escherichia coli* (EcMltC). We performed a comprehensive structural analysis to elucidate the conservation of the activity site and the unique features of the substrate binding groove. Our findings provide structural insights into the mechanism of peptidoglycan processing in this multidrug-resistant pathogen. Since LTs are essential for maintaining cell wall integrity, inhibiting their function can lead to bacteriolysis and potentiate the effect of other antibiotics. Therefore, elucidating the atomic resolution structure of AbMltC is not merely an expansion of the MltC family database, but a critical prerequisite for structure-based drug design. Understanding the specific structural features of AbMltC will provide a foundation for designing novel inhibitors to combat this critical pathogen.

## Materials and Methods

### Protein Preparation

Several N-terminus truncated MltC genes from *A. baumannii* were synthesized by BIONICS (Republic of Korea). Sequence information of full-length AbMltC gene was obtained at GenBank (Accession number: SST04976.1). The synthesized genes were individually inserted in a pET-21a vector using the *NdeI* and *XhoI* restriction sites. These expression constructs were then individually introduced into *E. coli* BL21 (DE3) host cells via a heat shock procedure at 42°C. Transformants were selected by plating on LB agar supplemented with ampicillin, followed by overnight incubation at 37°C. A single colony was selected and cultured in 10 ml of LB medium containing 50 μg/ml ampicillin. For large-scale protein production, the overnight culture was used to inoculate 1 L of LB medium. The culture was grown until the optical density at 600 nm reached a value between 0.6 and 0.8, at which point it was chilled on ice. Protein expression was then induced by adding 1 mM IPTG, and the cells were incubated with shaking overnight at 20°C.

Following protein expression, cells were harvested by centrifugation at 3,500 rpm (1,360 ×*g*) for 15 min at 20°C. The resulting cell pellet was resuspended in 10 ml of lysis buffer (20 mM Tris–HCl pH 8.0, 500 mM NaCl, 25 mM imidazole, and 0.1 mM PMSF). The cell suspension was sonicated on ice to disrupt the cells, and the resulting lysate was cleared of cell debris by centrifugation at 16,000 rpm (28,306 ×*g*) for 30 min at 4°C. The soluble fraction (supernatant) was collected and mixed gently with Ni-NTA resin (Qiagen, Germany) for two hours at 4°C to allow binding. The resin-protein mixture was then transferred to a gravity-flow column and washed with 30 ml of washing buffer (20 mM Tris-HCl pH 8.0, 500 mM NaCl, and 60 mM imidazole) to remove unbound proteins. The bound target protein was subsequently eluted from the column using 2.6 ml of elution buffer (20 mM Tris–HCl pH 8.0, 500 mM NaCl, 250 mM imidazole). Eluted fractions from each construct were analyzed by SDS-PAGE, and the construct exhibiting the highest level of overexpression was selected (containing residue 26–365). The corresponding overexpressed protein was then purified further through the next chromatography step. To achieve a higher level of purity, the eluted sample was further processed by size-exclusion chromatography (SEC) using an ÄKTA Explorer system (GE Healthcare, USA) with a Superdex 200 Increase 10/300 GL column (GE Healthcare). The column was pre-equilibrated with SEC buffer (20 mM Tris–HCl pH 8.0, 150 mM NaCl). The peak fractions containing the AbMltC protein were pooled and concentrated to a final concentration of 12.6 mg/ml. The concentrated proteins were flash-frozen in liquid nitrogen and stored at -80°C until further use.

### Crystallization and Data Collection

Initial crystal screening to find conditions for producing high-quality crystals was performed using the sitting-drop vapor-diffusion method at 20°C. The procedure involved combining 1 μl of the 12.6 mg/ml AbMltC sample with an equal volume of various reservoir solutions. Suitable crystals grew after 14 days in a solution of 60% (v/v) polypropylene glycol 400 and 0.1 M Tris-HCl pH 8.0 (from the Midas plus no. 37 screen, Molecular Dimensions). Before data collection, the crystals were briefly soaked in a cryoprotectant solution consisting of the mother liquor supplemented with 30% (v/v) glycerol. Diffraction data were then collected at a wavelength of 1.000 Å on the 5C beamline at the Pohang Accelerator Laboratory (PAL) in Pohang, Republic of Korea. Data processing was carried out using HKL-2000 [12].

### Structure Determination and Analysis

The structure was determined using the same methods as those applied in our previous study on the MltG structure [13]. Briefly, the molecular replacement (MR) phasing method was utilized to determine the protein structure, using the Phaser program within the PHENIX package [14]. The MR search model was generated based on the structural predictions from AlphaFold v2.0 via the ColabFold. Subsequent model building and refinement were conducted using Coot [15] and phenix.refine tools from the PHENIX package [16]. The quality of the final model was assessed using MolProbity [17]. Structural representations were generated using the PyMOL tool [18].

### SEC-Multi Angle Light Scattering (MALS) Analysis

To determine the absolute molar mass of the AbMltC protein in solution, multi-angle light scattering (MALS) analysis was conducted. The purified protein, which had been isolated by affinity chromatography, was passed through a 0.2 μm syringe filter and loaded onto a Superdex 200 10/300 gel-filtration column (GE Healthcare) that was pre-equilibrated with SEC buffer. The buffer flow rate was maintained at 0.6 ml/min at 25°C. A DAWN-TREOS MALS detector (Wyatt Technology, USA) connected to an ÄKTA Explorer system (GE Healthcare) was used to detect the scattered light. The ASTRA software (Wyatt Technology) was utilized to analyze the data and determine the absolute molecular mass.

### Sequence Alignment

The amino acid sequences of AbMltC and EcMltC were analyzed and aligned using the Clustal Omega bioinformatics tool, which is accessible at http://www.ebi.ac.uk/Tools/msa/clustalo/.

## Results

### The Overall Structure of AbMltC

**Fig. 1:**
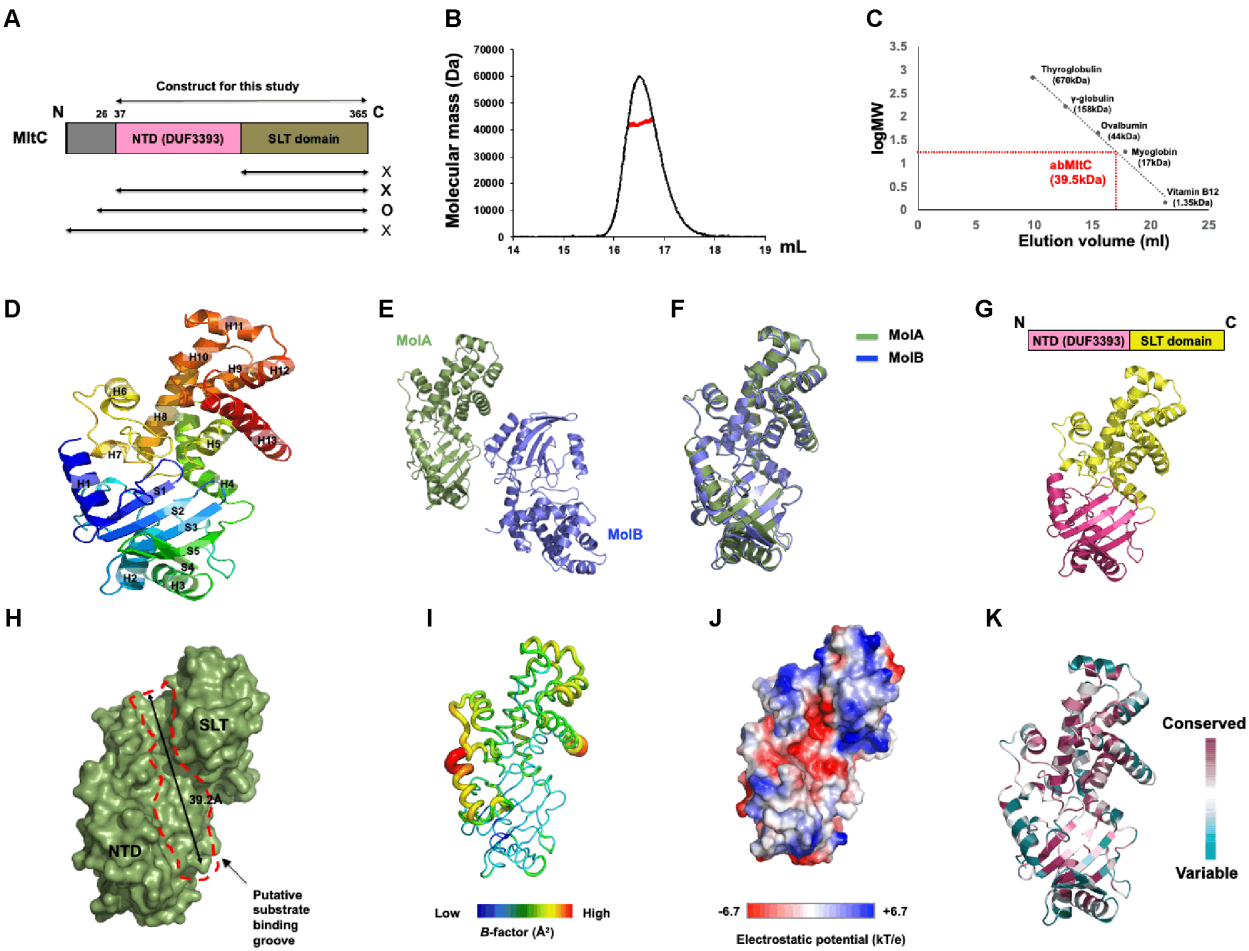
Overall crystal structure of AbMltC. (**A**) The schematic diagram showing the domain composition of AbMltC. The expression constructs that were used for expression test are indicated by black double arrows. O and X indicate “expressed” and “not expressed”, respectively. NTD and SLT domain indicate Nterminal domain and soluble lytic transglycosylase domain, respectively. (**B**) SEC-MALS profiles utilized in purifying AbMltC. Standard-size markers are shown above the profile, aiding in the determination of elution volumes. The experimental MALS data are represented by the red line, plotted against the SEC elution volume on the x-axis and the absolute molecular mass distributions on the y-axis. (**C**) The elution volume line fitting in SEC plotted against the size marker and the logarithm of the molecular weight of AbMltC. The red point on the fitting line signifies the elution volume of AbMltC. The molecular weights of the size markers are indicated along the standard line for reference. (**D**) The ribbon representation of AbMltC. The rainbow color scheme was used for tracing the N- to C-terminus. Helices and sheets are labeled with H and S, respectively. (**E**) Two AbMltC molecules detected in the same crystallographic asymmetric unit (ASU). (**F**) Structural superposition between two AbMltC molecules in the ASU. (**G**) Domain composition of AbMltC. (**H**) Surface representation of AbMltC. Putative substrate binding groove is indicated by red-dot line. (**I**) Putty representation conveying *B*-factor distribution. Rainbow colors from red to violet relative to *B*-factor values were used for *B*-factor visualization. (**J**) Electrostatic surface representation of AbMltC. The scale ranges from −6.7 kT/e (red) to +6.7 kT/e (blue). (**K**) Cartoon representation of AbMltC colored according to the degree of amino-acid sequence conservation analyzed by the Consurf server.

To investigate the structure of AbMltC, we first needed a soluble form of the protein. For this purpose, several expression vectors were generated to evaluate expression and solubility. As a result, an N-terminally truncated construct (residues 26–365), which lacks the cysteine involved in membrane linkage at the N-terminus, was successfully expressed in *E. coli* and confirmed to be soluble (Figs. 1A and S1). The target soluble AbMltC was purified via a rapid two-step chromatography process involving affinity chromatography and size exclusion chromatography (SEC) (Fig. 1B and 1C). SEC analysis indicated that AbMltC exists as a monomer in solution, eluting at approximately 16.5 ml between ovalbumin (44 kDa) and myoglobin (17 kDa). To more precisely determine the stoichiometry of MltC, we analyzed its absolute molecular mass using multi angle light scattering (MALS). The molecular weight of AbMltC in solution was measured to be 40.1 kDa (Figs. 1B, S2, and S3). Considering that the theoretical molecular weight of AbMltC is 39.6 kDa, these results confirm that AbMltC exists as a monomer in solution.

Monomeric AbMltC is composed of five β-strands and thirteen α-helices, which shows a compact architecture (Fig. 1D). Structural superposition of the two protein molecules present in the crystallographic asymmetric unit (ASU) revealed nearly identical conformations, with an RMSD of 0.8 Å (Fig. 1E and 1F). The protein displays the canonical two-domain architecture observed in other MltC, consisting of an N-terminal DUF3393 (Domain of Unknown Function) domain (NTD) and a C-terminal catalytic SLT (Soluble Lytic Transglycosylase) domain (Fig. 1G). Surface analysis revealed the presence of a deep groove, approximately 39.2 Å in length, located between the NTD and SLT domain, which is likely to represent the typical binding site for peptidoglycan (PG) substrate (Fig. 1H). *B*-factor analysis showed that this putative PG-binding region exhibited relatively low *B*-factors (around 48.2 Å2), indicating a structurally stable conformation, whereas the α-helices forming the N- and C-termini displayed comparatively higher *B*-factors (92.5 Å2) (Fig. 1I). Electrostatic surface potential analysis revealed a balanced distribution of positively charged, negatively charged, and non-polar residues. Notably, the region predicted to serve as the substrate-binding groove exhibited a negatively charged center flanked by positively charged and neutral areas at both ends (Fig. 1J). Finally, amino acid sequence conservation analysis using the ConSurf server [19] demonstrated that amino acids forming the putative substrate-binding pocket, including those constituting the active site, are highly conserved among homologous proteins, further supporting their functional importance (Fig. 1K).

### Structural Comparison between AbMltC and Its Structural Homologues

**Fig. 2:**
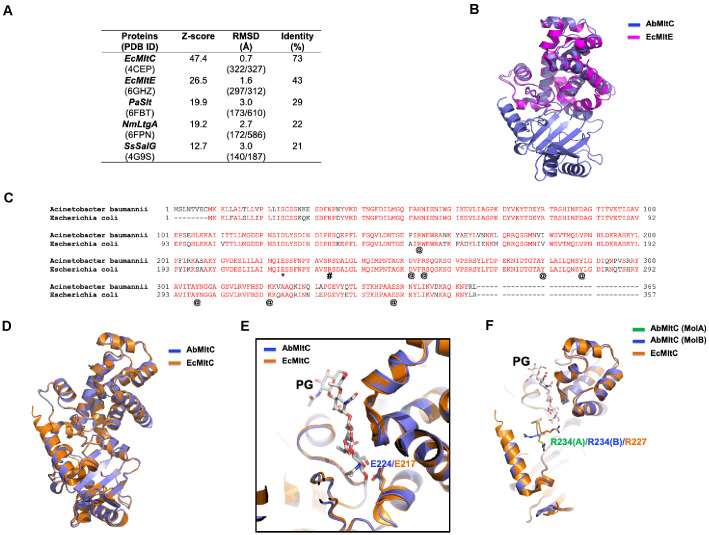
Structural comparison between AbMltC and its structural homologues. (**A**) Table summarizing the result of DALI search. Ec: *Escherichia coli*, Pa: *Pseudomonas aeruginosa*, Nm: *Neisseria meningitidis*, Ss: *Salmo salar*. (**B**) Pairwise structural superimposition of AbMltC (blue) with EcMltE (magenta; PDB ID: 6GHZ). (**C**) The sequence alignment of AbMltC with EcMltC. The residues, which were known to be the catalytic proton donor in the LTase reaction and to act as a “molecular ratchet” facilitating processive cleavage, were indicated by an asterisk (*) and hash (#), respectively. @ marks are key residues forming the substratebinding pocket identified in the EcMltC study, and they are fully conserved in AbMltC. Completely conserved residues are indicated by red. (**D**) Pairwise structural superimposition of AbMltC (blue) with EcMltC (orange; PDB ID: 4CFP). (**E**) Superimposed structures with a magnified view of the active site. PG indicates the peptidoglycan substrate. The glutamate residue acting as the nucleophile in the LTase reaction at the active site is labeled. (**F**) Structural comparison of residue R234, which is expected to act as a 'molecular ratchet' and contribute to facilitating processive cleavage.

Based on this observation, we employed the DALI server [20] to identify structural homologues of AbMltC. As expected, EcMltC was found to be the closest structural homologue, followed by MltE, Slt, and SalG, which represent a different LT family (Fig. 2A). These results indicate that, aside from its similarity to EcMltC, AbMltC shares considerable structural resemblance with the MltE family. To further explore this similarity, we superimposed the structures of AbMltC and EcMltE (Fig. 2B). Notably, EcMltE consists solely of an SLT domain, lacking the NTD present in AbMltC. Superposition of the SLT domains revealed a high degree of similarity, with an RMSD of approximately 0.9 Å, confirming that the SLT domain of AbMltC is structurally very similar to EcMltE.

AbMltC and EcMltC shared a high sequence identity of 73% (Fig. 2A and 2C). Structural alignment further confirmed their similarity, revealing an RMSD of 0.7 Å between the two structures (Fig. 2D). Structural alignment confirmed that E224 of AbMltC occupies an identical position to E217 in EcMltC, directly positioned toward the glycosidic oxygen of the substrate and known to be critical for the catalytic activity (Fig. 2E). The amino acid residues that form the substrate-binding pocket identified in the EcMltC study were found to be fully conserved in AbMltC (R153/R147, D251/D244, R254/R247, K321/K314, E348/E341, Y280/Y273, Y288/Y281, Y306/Y299) (Fig. 2C). Sequence analysis across *Gram*-negative MltC homologs shows strict conservation of this residue, supporting its essential catalytic role (Fig. 2C). In EcMltC, R227 was shown to be important for substrate binding and facilitating processive cleavage [9]. Sequence alignment indicates that AbMltC retains R234 at the equivalent position (Fig. 2C). However, in our structure, R234 adopts multiple side-chain conformations, suggesting increased flexibility of this residue (Figs. 2F and S4). This structural heterogeneity implies that substrate positioning or peptide stem recognition may be less rigid in AbMltC compared to EcMltC. Alternatively, in the case of MltC subfamily, the movement of R234 may be considered an important event during substrate processing. These structural comparisons suggest that while the core catalytic machinery is highly conserved across the Mlt family, local structural variations, particularly in the substrate-binding loops, may confer distinct substrate specificities or processivity characteristics to AbMltC.

## Discussion

Our study provides the first structural view of MltC from *A. baumannii*, complementing prior work on EcMltC. Since homologous proteins from different species may exhibit variations in both structure and mechanism, it is noteworthy that, unlike other members of the Mlt family whose structures and functions have been widely studied due to their critical roles in bacterial survival, EcMltC has remained the sole structural representative of the MltC subgroup. In this context, our determination of the MltC structure from *A. baumannii* represents the second MltC structure ever solved, underscoring its significance.

Structural comparison confirms that AbMltC preserves the characteristic two domain architecture (NTD and SLT) observed in EcMltC, which is essential for forming the extended substrate binding groove. However, while the PG-binding groove of EcMltC measures approximately 30 Å in length [9], accommodating about nine saccharide units, the corresponding groove in AbMltC extends to 39 Å, suggesting that it might be able to accommodate an additional 2–3 saccharide units.

Our structural data reinforces the indispensable catalytic role of the conserved E224, while the observed conformational flexibility of R234 distinguishes AbMltC from its homologs. In EcMltC, R227 has been suggested that it might undergo conformational shifts that enable processive advancement of the peptidoglycan chain [9]. The structural flexibility of R234 in AbMltC presents structural observations supporting the notion that this R234 undergo conformational movement to act as molecular ratchet. We propose that the conformational plasticity of R234 is functionally relevant the the enzymes processivity. For a MltC to cleave the peptidoglycan strand continuously, it must slide along the polymer chain. A rigid active site might hinder this translocation. the observed flexibility suggests that R234 functions as a dynamic ratchet, adjusting its confomation to accommodate the steric shifts of the backbone during translocation. This dynamic mode of action would allow AbMltC to maintain substrate affinity while permitting the stepwise movement required for efficient processive cleavage. While the precise functional implications remain to be tested biochemically, our findings highlight the flexibility of arginine residue near the active site for long substrate processing.

In conclusion, this study establishes the structural framework of AbMltC, revealing unique features distinguishable from its *E. coli* homolog. The identification of the extended substrate-binding groove and the mobile R234 residue highlights specific structural vulnerabilities that could be exploited for drug discovery. These findings not only advance our mechanistic understanding of bacterial LTs but also offer precise structural templates for the design of small molecule inhibitors aimed at weakening the cell wall of *A. baumannii*.

## Supplemental Materials

Supplementary data for this paper are available on-line only at http://jmb.or.kr.



## Figures and Tables

**Table 1:** Data collection and refinement statistics.