# Supplementary material for: Structural Insights into MltC from Acinetobacter baumannii: Conservation of the Catalytic Residue and Flexibility in Substrate Recognition
Source: J Microbiol Biotechnol. 2026 Jan 22;36:e2511019. doi: 10.4014/jmb.2511.11019 (PMC12861720; doi:10.4014/jmb.2511.11019)
Supplement: Supplementary file 1 [file jmb-36-e2511019-supple.pdf]

Structural insights into MltC from *Acinetobacter baumannii*: conservation of the catalytic residue and flexibility in substrate recognition

Hyunseok Jang[1], Chang Min Kim[2], and Hyun Ho Park[1,*]

[1]College of Pharmacy, Chung-Ang University, Seoul 06974, Republic of Korea.

[2]Department of Molecular Genetics, University of Texas Southwestern Medical Center, Dallas, TX 75390, USA.

[*]**Corresponding author:** Hyun Ho Park; College of Pharmacy, Chung-Ang University, Seoul 06974, Republic of Korea; Tel: +82-2-820-5930; Fax: +82-2-820-3033; Email: xrayleox@cau.ac.kr

>SST04976.1 transglycosylase [Acinetobacter baumannii]

MSLNTVECMK KLLALTLLVP LLISCSSNKE SDFNPNYVKD TNGFDILMGQ
FAHNIENIWG IKEVLIAGPK DYVKYTDEYR TRSHINFDAG TITVETISAV
EPSEHLKKAI ITTLLMGDDP NSIDLYSDIN DIPHSQEPFL FGQVLDNTGE
PIRWEWRANK YAEYLVNNKL QRRQSGMNVI WSVTMQLVPN HLDKRAHKYL
PFIRKASAKY GVDESLILAI MQIESSFNPY AVSRSDALGL MQIMPNTAGK
DVFRSQGKSG VPSRSYLFDP EKNIDTGTAY LAILQNSYLG DIQNPVSRRY
AVITAYNGGA GSVLRVFHSD KKVAAQKINQ LAPGEVYQTL STKHPAAESR
NYLIKVDKAQ KNYRL

Supplementary Fig. 1. **Sequence information of AbMltC**. Full amino acid sequence of AbMltC (GenBank: SST04976.1). The construct used in this study (residues 26-365) is colored in green.

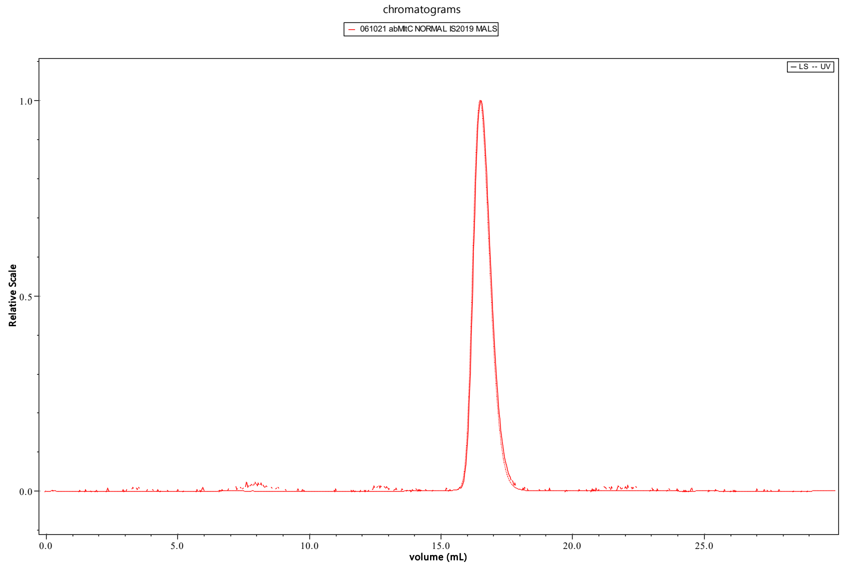

Supplementary Fig. 2. **MALS peak results of AbMltC.** The LS trace is represented by a solid line, and the UV trace is represented by a dashed line.

ASTRA 6 Report 061021 abMltC NORMAL IS2019 MALS

WYATT TECHNOLOGY

File Name: D:\Wyatt\Data\2021\061021 abMltC NORMAL IS2019 MALS.afe6
Collection Operator: END-2C345F10AD8\Administrator (END-2C345F10AD8\Administrator (admin))
Processing Operator: END-2C345F10AD8\Administrator (admin)

Sample: BSA
Concentration: 0.000 mg/mL

Configuration

Concentration Source: UV
Flow Rate: 0.500 mL/min

Light Scattering Instrument: TREOS
Band Broadening Correction: n/a
Cell Type: Fused Silica
Wavelength: 659.2 nm
Calibration Constant: 5.1380×10^{-5} 1/(V cm)

Detector	Scattering angle	Gain	Normalization coefficient
1	43.6°	n/a	0.741
2	90.0°	n/a	1.000
3	136.4°	n/a	0.725

UV Instrument: generic UV
Band Broadening Correction: Yes (Instrumental: 28.754 µL, Mixing: 91.577 µL)
UV Cell Length: 0.500 cm
UV Response Factor: 1.000 AU/V

Solvent: water
Temperature Correction Enabled: yes
Refractive Index: 1.331

Fluid Connections

Source Instrument	Destination Instrument	Delay Volume (mL)
Generic Pump	Injector	0.000
Injector	Generic UV Instrument	0.000
Generic UV Instrument	miniDAWN TREOS	0.164

Aux Connections

Source Instrument	Destination Instrument	Aux Channel	Calibration Constant
Generic UV Instrument	miniDAWN TREOS	1	1.000

Processing

Collection Time: Thursday June 10, 2021 01:23:06 PM ´ëÇÑ¹Î±¹ Ç¥ÁØ½Ã
Processing time: Friday December 19, 2025 09:06:30.296 AM ´ëÇÑ¹Î±¹ Ç¥ÁØ½Ã

Basic Collection:
LS Instrument Collection Interval: 1.000 sec

Baselines:

ASTRA 6.0.6.13 Page 1 of 2

ASTRA 6 Report 061021 abMltC NORMAL IS2019 MALS

Baselines:

Series	Start	Stop	Type
detector 1	(0.472, 0.074)	(62.111, 0.075)	manual x, auto y
detector 2	(0.472, 0.022)	(62.111, 0.022)	manual x, auto y
detector 3	(0.472, 0.043)	(62.111, 0.043)	manual x, auto y
channel	(0.472, 0.049)	(62.111, 0.049)	manual x, auto y

Peak settings:

**Peak Name**	Peak 1
**Peak Limits (min)**	32.532 - 33.484
**Light Scattering Model**	Zimm
**Fit Degree**	1
**dn/dc (mL/g)**	0.1850
**A2 (mol mL/g²)**	0.000
**UV Ext. Coef. (mL/(mg cm))**	1.239

Results Fitting Procedure:

Data	Fit Model	Degree	R^2	Extrapolation
Molar Mass	none	n/a	n/a	none
rms radius	none	n/a	n/a	none
mean square radius	none	n/a	n/a	none

Results

Peak Results

	Peak 1
**Masses**	
**Injected Mass (µg)**	0.00
**Calculated Mass (µg)**	373.16
**Mass Recovery**	n/a
**Mass Fraction (%)**	100.0
**Molar mass moments (g/mol)**	
**Mn**	4.252×10^{4} (±1.818%)
**Mp**	4.227×10^{4} (±0.986%)
**Mv**	n/a
**Mw**	4.253×10^{4} (±1.796%)
**Mz**	4.254×10^{4} (±3.996%)
**Mz+1**	4.255×10^{4} (±6.408%)
**M(avg)**	4.281×10^{4} (±0.074%)
**Polydispersity**	
**Mw/Mn**	1.000 (±2.555%)
**Mz/Mn**	1.000 (±4.390%)
**rms radius moments (nm)**	
**Rn**	14.2 (±27.9%)
**Rw**	14.2 (±28.0%)
**Rz**	14.1 (±28.0%)
**R(avg)**	11.2 (±1.8%)

Supplementary Fig. 3. **Details of SEC–MALS analysis of AbMltC.**

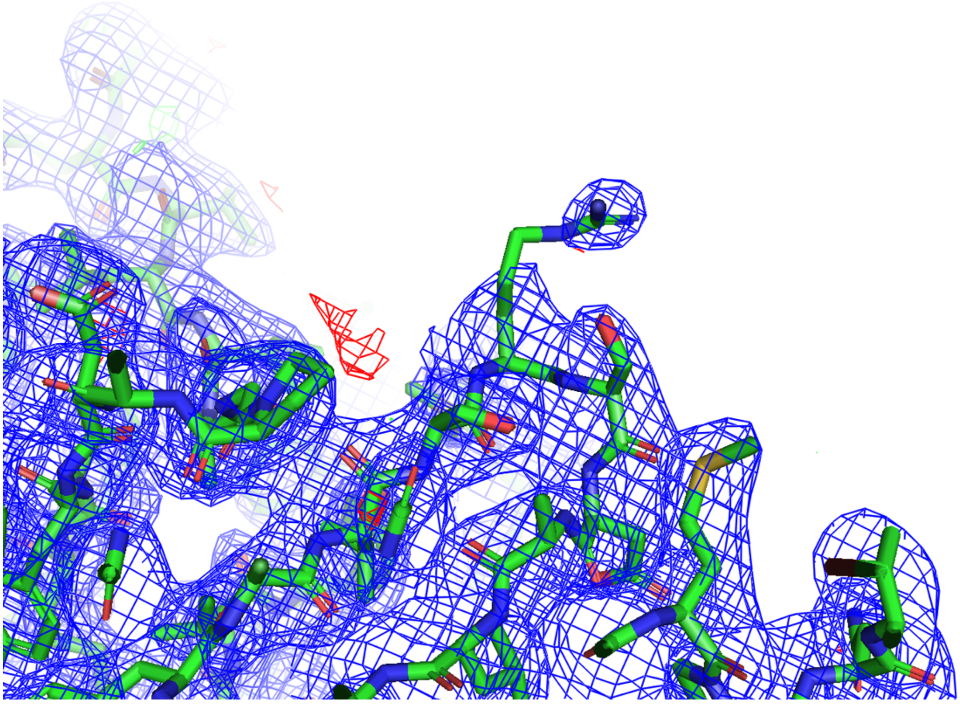

Supplementary Fig. 4. Close-up view of residue R234 region. 2Fo−Fc electron density map contoured at the 1σ level around R234 is indicated by the blue mesh.
